# Age-Related Changes in Sleep and Circadian Rhythms: Impact on Cognitive Performance and Underlying Neuroanatomical Networks

**DOI:** 10.3389/fneur.2012.00118

**Published:** 2012-07-26

**Authors:** Christina Schmidt, Philippe Peigneux, Christian Cajochen

**Affiliations:** ^1^Centre for Chronobiology, Psychiatric Hospital of the University of BaselBasel, Switzerland; ^2^Neuropsychology and Functional Neuroimaging Research Unit, Université Libre de BruxellesBruxelles, Belgium

**Keywords:** aging, sleep-wake regulation, cognition, functional magnetic resonance imaging, circadian rhythms, sleep homeostasis

## Abstract

Circadian and homeostatic sleep-wake regulatory processes interact in a fine tuned manner to modulate human cognitive performance. Dampening of the circadian alertness signal and attenuated deterioration of psychomotor vigilance in response to elevated sleep pressure with aging change this interaction pattern. As evidenced by neuroimaging studies, both homeostatic sleep pressure and circadian sleep-wake promotion impact on cognition-related cortical and arousal-promoting subcortical brain regions including the thalamus, the anterior hypothalamus, and the brainstem locus coeruleus (LC). However, how age-related changes in circadian and homeostatic processes impact on the cerebral activity subtending waking performance remains largely unexplored. Post-mortem studies point to neuronal degeneration in the SCN and age-related modifications in the arousal-promoting LC. Alongside, cortical frontal brain areas are particularly susceptible both to aging and misalignment between circadian and homeostatic processes. In this perspective, we summarize and discuss here the potential neuroanatomical networks underlying age-related changes in circadian and homeostatic modulation of waking performance, ranging from basic arousal to higher order cognitive behaviors.

## Introduction

Aging can be defined in terms of life and time (Martin, 1981) and it is often assumed that cognitive and health difficulties tend to increase as time advances. However, many researchers depart from this stereotype and put the concept of successful aging forward (for a review, see Lupien and Wan, 2004). Aging is considered as a multidimensional process in a way that environmental factors may protect for or conversely aggravate signs of aging in a non-linear manner with regard to physiological but also neurobehavioral processes. There is large and important heterogeneity both in cognitive and sleep-wake rhythm alterations that occur with normal aging, which have the potential to serve as a tool to better understand its underlying processes (Rowe and Kahn, 1987; Lupien and Wan, 2004; Eyler et al., 2011).

In the 1970s and 1980s, coupled oscillator models were shown to reproduce the basic features of the timing of human sleep and wake episodes, with one oscillator representing sleep/wake and the other representing the circadian pacemaker driving the temperature cycle (Wever, 1975; Kawato et al., 1982; Kronauer et al., 1982). Alternatively, the two process model of sleep regulation has been put forward at the same time by Borbely (1982) and Daan et al. (1984), and based on these models it was recently shown that a physiologically based model is able to account for many features of human sleep on self-selected schedules (Phillips et al., 2011). In this review we refer to the two process model, relying on a fined tuned interaction between the sleep-wake homeostatic and the circadian process to allow maintenance of sleep and wakefulness at appropriate times of day in order to explain time of day modulations in subjective sleepiness and cognitive performance.

There is ample evidence that the interplay of circadian and homeostatic processes also determines the temporal modulation of sleepiness and alertness levels across the day, which in turn affects performance for different cognitive domains (Cajochen et al., 2004; Dijk and von Schantz, 2005; Dijk and Archer, 2009). Disturbances or imbalance in the relationship between the circadian and homeostatic systems can lead to sleep and/or mood disorders and major difficulties in maintaining optimal cognitive performance during wake time.

Even in the absence of clinically significant sleep disorders, healthy aging is associated with a decline in night-time sleep quality and duration, decreases in sleep depth, sleep intensity, and sleep continuity (Bliwise, 2005). Concomitantly, a reduced amplitude of circadian rhythm output signals has been shown in older participants (Dijk et al., 1999; Duffy and Czeisler, 2002; Münch et al., 2005), suggesting that age-related changes in sleep may be partially due to a weaker circadian regulation of sleep and wakefulness. In parallel, it has been observed that older people may need less sleep (Klerman and Dijk, 2008) suggesting that in spite of marked changes in sleep physiology, excessive daytime sleepiness is not common during healthy aging (Duffy et al., 2009).

The underlying cerebral mechanisms of homeostatic and time of day-dependent modulation patterns in cognitive performance remain largely unexplored, in particular in relation to the healthy aging process. Recent functional magnetic resonance imaging (fMRI) studies in young volunteers yielded evidence that this interaction also influences cognition-related cortical (mainly frontal) and subcortical (thalamic, hypothalamic, and brain stem locus coeruleus, LC) brain activity (Schmidt et al., 2009, 2012; Vandewalle et al., 2009). Furthermore, there is evidence that cortical and subcortical task-related BOLD activity declines in those individuals presenting higher vulnerability to sleep loss and circadian misalignment while it increases in those participants who are less susceptible (e.g., Chuah et al., 2006; Vandewalle et al., 2009).

Similar fMRI studies are not yet available in older individuals. However, post-mortem studies revealed neuronal loss in the SCN of older people (Hofman and Swaab, 2006). Also, neuron density within the LC decreases with age due to a progressive loss of noradrenergic neurons, both in animals and humans (Samuels and Szabadi, 2008). Furthermore, the number of LC neurons projecting to areas such as the frontal cortex and the hippocampus declines with age, resulting in fewer synapses (Samuels and Szabadi, 2008). Since the LC is also involved in the regulation of cognitive performance (Usher et al., 1999), it can be hypothesized that age-related changes in these arousal-promoting structures may crucially contribute to circadian-related alterations in cognitive abilities. At the cortical level, frontal brain regions are particularly prone to both the aging process and to the misalignment between circadian and homeostatic processes, even though recent evidence indicates dissociation between these influences on frontal-activation-related executive functions (Cain et al., 2011; Tucker et al., 2011; Bratzke et al., 2012).

In this review, we will discuss the influence of circadian and homeostatic regulation on waking performance, including recent insights into the underlying cerebral correlates of the observed behavioral modulations. The impact of the age factor on these brain networks will then be discussed, considering that it is most likely that cognitive decline is a multifactorial process and that reserve factors may compensate for age-related modifications both in sleep features and cognitive functions (Bartres-Faz and Arenaza-Urquijo, 2011).

## Cerebral Correlates Underlying Circadian and Homeostatic Regulation of Waking Performance throughout the 24-h Cycle

**Figure 1:**
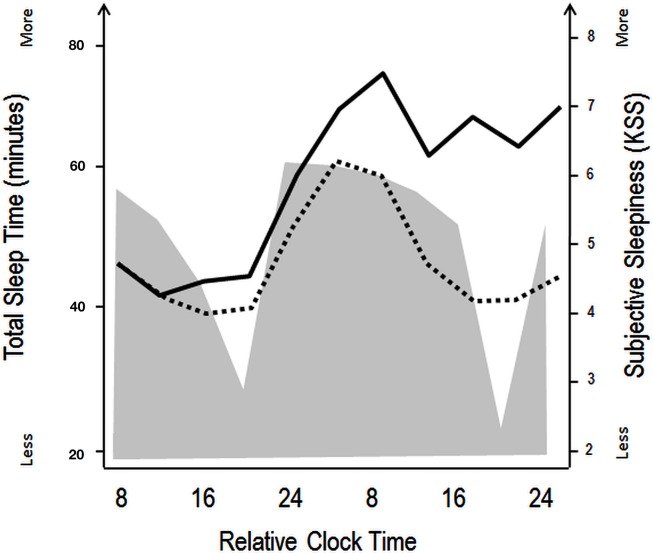
**Schematic illustration of the impact of circadian and homeostatic processes on sleep and wakefulness**. The filled gray area illustrates variations in total sleep time during a constant routine protocol with regularly occurring naps (150 min of wakefulness followed by 75 min of naps), aiming at investigating circadian rhythm parameters under low homeostatic sleep pressure conditions. Black lines indicate superimposed subjective sleepiness as assessed by the Karolinska Sleepiness Scale over a similar nap (dashed line) and total sleep deprivation (straight line) protocol. The wake maintenance zone can be identified in the naps scheduled in the subjective evening hours, with minimal total sleep time (expressed in minutes). The sleep-promoting signal in the biological night is accompanied by rapid increases in subjective sleepiness in both the low (naps) and high (sleep deprivation) sleep pressure conditions. Over the course of the second biological day, subjective sleepiness decreases, even when homeostatic sleep pressure increases (in the sleep deprivation protocol, straight line), indicating that circadian wake promotion rises or that circadian sleep promotion diminishes [modified from Cajochen et al. (2001) and Münch et al. (2005)].

The specific timing and consolidation of sleep and wake episodes within the 24-h light-dark cycle are regulated by a coordinated action of homeostatic and circadian processes (Borbely, 1982; Daan et al., 1984; see also Figure 1). It is assumed that the circadian and homeostatic process represent independent drives on sleep-wake propensity but interact in a non-linear fashion across the 24-h light-dark cycle. Thus, circadian-based wake propensity is at its highest levels during the early evening hours (commonly after 12 h of wakefulness), when homeostatic sleep pressure is rather high, whereas circadian propensity for sleep reaches its maximum during the early morning (∼2 h before habitual wake up time), when homeostatic sleep pressure is low (Dijk and Czeisler, 1994). At any given time, the magnitude of sleepiness, alertness, and fatigue is thus determined by the interacting influences of these two processes (Figure 1). After homeostatic sleep pressure has mostly dissipated over the first hours of the night, it is the high circadian-based propensity for sleep that prevents us from prematurely waking up in the early morning hours. Conversely, it is the very low circadian-based propensity for sleep (i.e., circadian wake-promoting signal) that prevents us from falling asleep in the early evening hours when homeostatic sleep pressure is at its highest level. In both cases, circadian and homeostatic systems ideally work in opposition to ensure a consolidated period of sleep or wakefulness (Dijk and Czeisler, 1994, 1995; Dijk and von Schantz, 2005).

The impact of the circadian timing system goes beyond compelling the body to fall asleep and to wake up again (Blatter and Cajochen, 2007; Schmidt et al., 2007; Wright et al., 2012). Forced desynchrony, constant routine, and sleep deprivation studies have identified the respective contributions of homeostatic sleep pressure and circadian rhythmicity on neurobehavioral performance measures (Dijk et al., 1992; Johnson et al., 1992; Cajochen et al., 1999, 2004; Wyatt et al., 1999; Carrier and Monk, 2000; Horowitz et al., 2003; Rogers et al., 2003). Two important observations have been made from these controlled studies: (1) while performance deterioration is mostly seen when the wake episode is extended into the biological night, modulations can also be seen throughout a usual waking day episode (<16 h of wakefulness) which can lead to significant deteriorations in the cognitive output and (2) the observed effects of the circadian and sleep-wake homeostatic system do not simply add up to characterize daily performance modulations. In particular, the circadian amplitude of cognitive performance modulation clearly depends on homeostatic sleep pressure levels (Dijk and Franken, 2005).

The proposal mentioned above that circadian and homeostatic systems interact at the neurobehavioral level has been supported by anatomical findings. In terms of circadian sleep-wake regulation, the SCN is the central circadian pacemaker regulating sleep-wake timing. The SCN sends an indirect projection – relayed via the dorsomedian hypothalamus – to the noradrenergic LC, which in turns sends wide projections to the entire cortex (Aston-Jones et al., 2001; Aston-Jones, 2005). Consequently, the LC has been proposed to be implicated in the circadian regulation of higher order cognitive behaviors (Gompf and Aston-Jones, 2008). On the other hand, the cerebral correlates and exact anatomical location of the sleep homeostat are still unknown. It most likely represents a diffuse system that includes the accumulation of at least one sleep-promoting substance, which enhances the activity of sleep-promoting, and reduces the activity of wake-promoting neurons (Landolt, 2008). Accordingly, sleep homeostasis has been related to plastic processes occurring during wakefulness that result in a net increase in synaptic strength in many brain circuits (Tononi and Cirelli, 2003). From this perspective, sleep would serve to downscale synaptic strength to a baseline level that is energetically sustainable with the aim of a homeostatic regulation of the total synaptic weight impinging on neurons.

**Figure 2:**
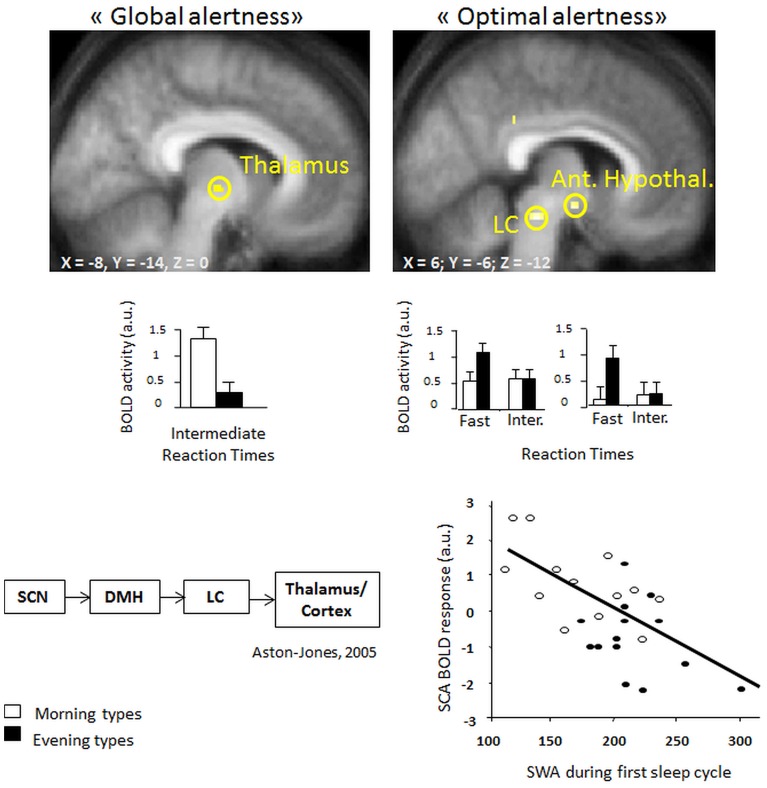
(Left, top panel) higher task-related thalamic activation in morning as compared to evening types for intermediate reaction times (“global alertness”) during the subjective evening hours [modified from Schmidt et al. (2009)] (right, top panel) Higher BOLD activity in locus coeruleus and anterior hypothalamic regions in evening as compared to morning types for “optimal alertness” (10% of fastest reaction times, as compared to intermediate reaction times). (Left, bottom panel) both regions have been implicated in circadian arousal regulation, as illustrated by the model of Aston-Jones et al. (2001). (right, bottom panel) Finally, optimal alertness-related activity in the anterior hypothalamus (i.e., suprachiasmatic area) is negatively related to the amounts of EEG slow wave activity at the beginning of the night, which can be considered as a reliable marker of homeostatic sleep pressure build-up [modified from Schmidt et al. (2009)].

Recent evidence from fMRI investigations in young morning and evening chronotypes indicate that homeostatic sleep pressure exerts an influence on attention-related cerebral activity in anterior hypothalamic structures, putatively implicated in the regulation of the circadian wake-promoting signal (Schmidt et al., 2009; see Figure 2). In particular, maintenance of optimal attentional performance in a vigilance task (PVT; psychomotor vigilance) after accumulated sleep pressure (i.e., during the subjective evening) was associated with higher activity in evening than morning chronotypes in the LC and in the anterior hypothalamus, two key structures crucially involved in the generation of the circadian wake-promoting signal. Furthermore, activity in the anterior hypothalamus decreased with increasing homeostatic sleep pressure as indexed by electroencephalographic (EEG) slow wave activity [SWA; EEG power density during non-rapid eye movement (Non-REM) sleep in the range of 0.75–4.5 Hz] in the first sleep cycle, suggesting that homeostatic and circadian interactions influence the neural activity underpinning diurnal variations in human behavior. Interestingly, this activation pattern was observed solely for the 10% of fastest reaction times that reflect the phasic ability to recruit the attentional network above normal levels (Drummond et al., 2005a).

Recently, a 24-h sleep deprivation study (Vandewalle et al., 2009) took advantage of a genetic trait (the hPER3 polymorphism; Viola et al., 2007) associated with differential vulnerability to the deleterious effects of sleep deprivation on neurobehavioral performance. This study revealed that from the morning (1.5 h of wakefulness) to the evening (14 h of wakefulness) of a normal waking day, the more resistant PER3^4/4^ individuals did not exhibit significant changes in brain responses to a working memory task, whereas the more vulnerable PER3^5/5^ participants presented decreased activity in the posterior dorso-lateral prefrontal cortex. When further challenging the sleep homeostat by 25 h of total sleep deprivation, the more vulnerable PER3^5/5^ subjects presented various decreased task-related cortical activations in the morning after sleep loss. In contrast, *PER3^4/4^* still did not show decreased brain responses to the task, but rather recruited supplemental brain areas located in right inferior frontal, middle temporal, parahippocampal gyri, as well as in bilateral thalamic areas. Similarly, morning types, more vulnerable to the accumulation of time spent awake throughout a normal waking day (Kerkhof, 1991; Mongrain et al., 2006a,b) show decreased BOLD responses in brain areas involved in conflict resolution over a normal waking day while performing the Stroop paradigm (Schmidt et al., 2012). In contrast, evening chronotypes, less affected by accumulated homeostatic sleep pressure during the evening exhibited the reversed profile or presented stable BOLD responses from morning to evening hours in task-related brain regions (Schmidt et al., 2012).

## Age-Related Modulation in Circadian and Homeostatic Regulation of Sleep and Waking Performance

**Figure 3:**
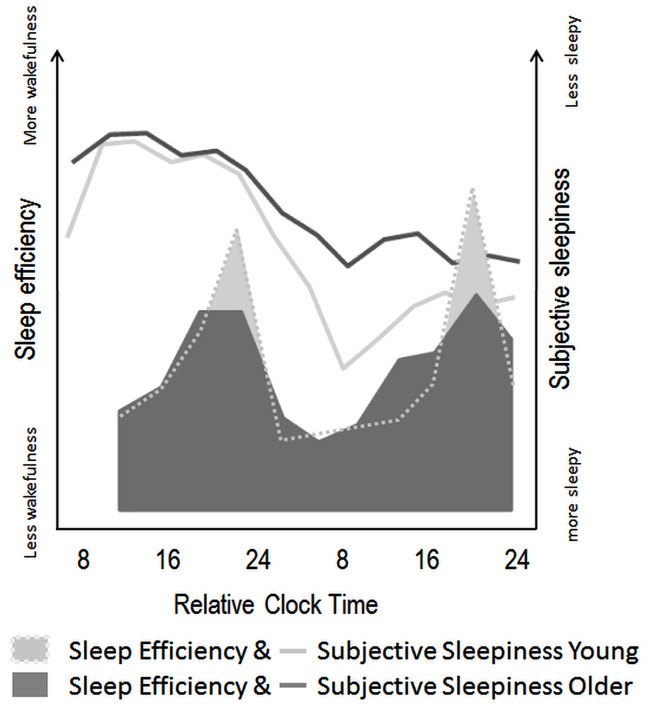
**Schematic illustration of age-related modifications in circadian and homeostatic sleep-wake regulation in humans**. Filled areas illustrate variations in sleep efficiency over a nap protocol (10 episodes of 150 min of wake, followed by 75 min of scheduled sleep episodes) modified from Münch et al. (2005). Circadian sleep-wake promotion, as expressed by the amount of wakefulness throughout nap episodes seems attenuated in older (dark gray) as compared to young individuals (light gray area). Line plots indicate the superimposed time course of subjective sleepiness over a 40-h sleep deprivation protocol in young (light gray line) and older (dark gray line) adults [modified from Adam et al. (2006)]. These values indicate less pronounced effects of increasing homeostatic sleep pressure on subjective sleepiness in older, as compared to young individuals.

It has been controversial whether age-related sleep changes result from alterations in circadian and homeostatic processes or in their precise interaction (see Figure 3 for a schematic illustration of age-related changes on circadian and homeostatic sleep-wake regulation). The age-related decline in absolute levels of slow wave sleep (SWS) represents one of the most common reported features in the ageing and sleep literature (Bliwise, 2005). Studies demonstrated that older adults respond to sleep loss with an increase in EEG SWA (Dijk et al., 2001) indicating that, even though older persons present lower absolute SWS levels, the homeostatic response to increasing sleep need is basically operational. However, older adults also showed a shallower decline in homeostatic sleep pressure after sleep deprivation, particularly in frontal brain regions (Dijk et al., 1989; Münch et al., 2004). Together with the recently observed age-related reduction in asymptotic sleep duration under extended sleep conditions, these data favor the assumption that older adults have a generally lower homeostatic need for sleep (Klerman and Dijk, 2008). In the same perspective, healthy aging was associated with a reduction in daytime sleep propensity, while sleep continuity and SWS were reduced (Dijk et al., 2010).

From a circadian perspective, older adults present a reduced amplitude of circadian rhythmicity in endogenous core body temperature (Dijk and Duffy, 1999) and melatonin (Münch et al., 2005), suggesting that age-related changes in sleep can also be related to a weaker circadian regulation. Whether age merely affects the wake- or sleep-consolidating function of the circadian signal has been a topic of debate. Dijk and colleagues found evidence that sleep latencies were rather similar between age groups throughout the circadian cycle, even though the shortest sleep latency values located around the temperature nadir were slightly longer in older participants (Dijk and Duffy, 1999; Dijk et al., 1999). Concomitantly, Duffy et al. (1998) reported that sleepiness and alertness levels in the older were less affected than in young adults, when the scheduled wake period occurred in the early morning hours coinciding with the maximal circadian drive for sleep. Finally, a nap study revealed that the circadian wake-promoting signal in the evening hours was weaker in older participants, with higher subjective sleepiness ratings and more sleep occurring during the wake maintenance zone in the late afternoon (Strogatz et al., 1987) in older than in young adults (Münch et al., 2005). Thus, the age-related lower homeostatic sleep need may account for the observed less consolidated and shorter sleep during night-time, while reduced circadian wake promotion during the biological day might favor daytime naps in older adults.

Cross-sectional studies indicate a preference for earlier habitual bedtime and getting-up time in older adults as compared to younger individuals (Carrier et al., 1997; Duffy et al., 1998; Duffy and Czeisler 2002). This morningness preference has been associated with an advance in the circadian phase at the physiological level, which could theoretically be associated to differences in the intrinsic period of the circadian oscillator (Brown et al., 2011). In this perspective, Pagani et al. (2010) showed proportionality between the physiological period length of the human circadian clock *in vivo* and the period in human fibroblasts in young and older participants. Interestingly, measurement of human fibroblasts in the presence of human serum from older donors highlighted shortened period length and advanced phase of cellular circadian rhythms as compared with serum from young donors, indicating that a circulating factor might alter human chronotype (Pagani et al., 2011). However, *in vivo* under conditions of experimentally induced misalignment between the sleep-wake cycle and endogenous circadian rhythmicity, the investigation of the circadian period in melatonin secretion or core body temperature revealed very similar period lengths across age groups (Czeisler et al., 1999; Duffy and Czeisler, 2002). However, the phase angle of entrainment as indexed by the timing of the biological clock (i.e., circadian phase) in relation to the timing of sleep (i.e., usual bedtime) was different in young and older participants: while young morning types woke up later within their circadian cycle (i.e., longer phase angles; e.g., Duffy et al., 1999; see Emens et al., 2009 for naturalistic conditions), older morning types woke up earlier within the circadian cycle (i.e., shorter phase angles; Duffy et al., 1999).

These age-related alterations in circadian and homeostatic sleep regulation significantly impact on an individual’s daytime cognitive performance level. Thus, from a clinical point of view, taking time of day and the individual’s circadian preference into account when assessing cognitive functions across age groups is rather important and has been emphasized in a series of reports (Hasher et al., 2005). Indeed, studies carried out under normal day–night conditions have generally revealed that, whereas the cognitive performance of young evening type adults often improves over the day, old morning type adults markedly deteriorate (May et al., 1993, 2005; Yoon, 1997; May and Hasher, 1998; Hasher et al., 1999, 2002, 2005; May, 1999; Yoon et al., 2003; Schmidt et al., 2007; Yang et al., 2007). This effect has been referred to as the synchrony effect, or the beneficial impact of temporal matching between task timing and preferred time of day for diurnal activities (May et al., 1993). The synchrony effect applies to different cognitive domains, including short-term memory tasks such as word span measures (Yoon, 1997), performance on different long-term memory tasks (May and Hasher, 1998; Intons-Peterson et al., 1999; Winocur and Hasher, 2002), and executive functions, especially cognitive inhibition abilities (Intons-Peterson et al., 1998; May and Hasher, 1998; May, 1999; West et al., 2002). We have recently observed that adapting testing time according to the specific individual’s sleep-wake schedule can attenuate synchrony effects in PVT and Stroop tasks (Schmidt et al., 2012), suggesting that part of the reported synchrony effects in aging may be accounted for by a series of confounders (e.g., differences in socio-professional timing constraints, the amount of accumulated sleep need or circadian phase position, all modulating arousal level at testing) rather than being inherent to the chronotypical profile of an individual. In the same vein, time of season may also affect cognitive functions, especially in clinical populations, such as bipolar I disorder (Rajajarvi et al., 2010). In the healthy population, there are indications that seasonal variation in mood can impact on cognitive performance (Merikanto et al., 2011).

Additionally, it is worth noting that the extent of age-related changes in circadian and sleep physiology substantially differs between individuals. For instance, the above mentioned polymorphism in the human clock gene PER3 may contribute to inter-individual differences in sleep and circadian physiology in older people (Viola et al., 2012). Homozygosity for the longer allele (PER3^5/5^) was associated with a phase-advance in the circadian melatonin profile and an earlier occurrence of the melatonin peak within the sleep episode. Furthermore, older PER3^5/5^ participants accumulated more nocturnal wakefulness, had increased EEG frontal delta activity (0.75–1.5 Hz), and decreased EEG frontal sigma activity (11–13 Hz) during (Non-REM) sleep compared with PER3^4/4^ participants.

Finally, when looking at the impact of circadian and homeostatic modulations on neurobehavioral performance in aging, weaker variations in circadian output measures were observed for both subjective and objective vigilance measures in older study participants. Declines in PVT performance seem less susceptible to circadian and homeostatic misalignment in older people (Blatter et al., 2006). Intriguingly, a reversal of age-related differences in PVT performance speed has been reported when testing was scheduled to the early morning hours during SD, i.e., when elevated homeostatic sleep pressure and minimal circadian wake promotion coincide, such that the older reacted faster than the young participants (Adam et al., 2006; Duffy et al., 2009). More recently, a forced desynchrony study similarly revealed that response speed on a sustained attention task and the ability to perform mental arithmetic are less deteriorated by the cumulative effects of repeated exposure to adverse circadian phase in older as compared to young individuals (Silva et al., 2010). In the same line, during a sleep fragmentation protocol, older participants were less sensitive to the imposed sleep disturbance in terms of performance decrements than young participants (Bonnet, 1989). Together, these data suggest an age-related attenuation of the wake-dependent homeostatic influence on cognitive performance (Silva et al., 2010). Alternatively, weakening of the circadian signal promoting wakefulness in the late biological day, also called the wake maintenance zone, may be responsible for the observed effects (Cajochen et al., 2006). However, in the above mentioned study (Silva et al., 2010), it was found that older participants had fewer lapses of attention in the circadian phase bins corresponding to the late biological day/early biological evening. It is also worth noting that optimal reaction time performance (i.e., the fastest 10% reaction times in the PVT) revealed that neither the wake-dependent homeostatic nor the circadian influence showed a significant interaction with age, suggesting that for both age groups, optimal reaction time performance is affected similarly by wake-dependent and circadian influences.

## Cerebral Underpinnings of the Circadian and Homeostatic Regulation of Waking Performance: Age-Related Influences?

Overall, more disruption in sleep and circadian rhythm outputs have been linked to increased disease susceptibility (Hastings et al., 2003), which both occur more often with advanced age. However, with increasing age, circadian, and sleep-wake related neural areas or the connections within the functional neuroanatomical networks may compensate for initial dysfunction (van Someren et al., 2002).

Hypothalamic dysfunction may potentially trigger some age-related physiological and behavioral changes in sleep-wake patterns, but the effects of senescence on specific hypothalamic nuclei that mediate these alterations have still to be elucidated (Kessler et al., 2011). Even though controversial, post-mortem studies indicate neuronal degeneration of the SCN in senescence, which suggests that the circadian pacemaker in the human brain becomes progressively disrupted during aging (Hofman and Swaab, 2006). Although no age-related changes were found in the number or size of SCN cells in the rhesus (Roberts et al., 2012), changes in spontaneous SCN neuron firing activity have been reported in aged rodents, together with alterations in the expression of certain genes and peptides similarly to findings in non-human primates (see Bertini et al., 2010 for a review). Also, Nakamura et al. (2011) reported reduced amplitude of day–night differences in neural activity with increasing age in mice, together with an alteration in neural activity in the subparaventricular zone, one of the main neural outputs of the SCN. As parallel studies indicate that the molecular clockwork in the SCN as measured by PER2 exhibits only minor deficits at the same age of those presenting reduced day–night amplitude and neural activity in the subparaventricular zone, it is suggested that the circadian output measured at the level of neural activity rhythms in the SCN is degraded by aging, before disruption becomes evident in key components of the molecular clockwork (Roberts et al., 2012).

Circadian arousal regulation acts via indirect projections from the SCN to the arousal-promoting LC in the brainstem (Aston-Jones et al., 2001). Interestingly, the LC has the potential to impact on higher order cognitive performance and shows early vulnerability to the aging process, such that neuron density within the LC decreases with age due to a progressive loss of cell number and size of noradrenergic neurons both in animals and humans (Samuels and Szabadi, 2008). Furthermore, the loss of noradrenergic LC axons innervating the frontal cortex has been associated with modifications in the electrophysiological properties of the remaining LC terminals (Samuels and Szabadi, 2008). The SCN has also a weak direct projection to the wake-active orexin-producing neurons in the lateral hypothalamic area (Saper et al., 2001). Since the densest projection of orexin fibers terminates in the LC, it has also been suggested that the LC controls the activity of orexin neurons directly by inhibiting orexin firing and indirectly via the DMH. Interestingly, orexin secretion follows a circadian variation in rats (Zhang et al., 2004), monkeys (Zeitzer et al., 2003), and humans (Salomon et al., 2003), which might be the result of direct or indirect inputs from the SCN to the orexin circuits. Indeed lesions of the SCN suppress the daily orexin rhythm (Deboer et al., 2004; Adamantidis and de Lecea, 2008). On the other side, orexin levels increased in response to sleep deprivation in both control and SCN-lesioned animals, demonstrating that sleep homeostatic control of orexin occurs independently from the SCN (Deboer et al., 2004). It is known that orexin in the lateral hypothalamus influences many integrative homeostatic processes related to wakefulness and plays a crucial role in sleep architecture and state stabilization throughout the sleep-wake cycle (Saper et al., 2001). Kessler et al. (2011) observed that aged rats exhibited a loss of greater than 40% of orexin-immunoreactive neurons, suggesting that compromised orexin function could be an important mediator of age-related homeostatic disturbances of hypothalamic origin.

The cholinergic basal forebrain represents an additional important key area in the regulation of wakefulness-related cortical arousal that is selectively sensitive to both prolonged waking and aging (Cayetanot et al., 2005). Interestingly, the sleep-wake-dependent decline and rise in adenosine levels, a potential sleep-promoting substance, is more pronounced in the basal forebrain than in other cerebral regions (Strecker et al., 2000). Accordingly, it has been suggested that local release of adenosine in the basal forebrain provides a signal for the homeostatic regulation of Non-REM sleep (Landolt, 2008). Furthermore, age-related attenuation in basal forebrain function reduces its capacity to respond to increased neuronal activity during prolonged wakefulness (Cayetanot et al., 2005). In a similar perspective, cognitive stimulation in humans increases lactate expression in the prefrontal cortex of young, but not in an aged population (Urrila et al., 2004).

All of these cerebral systems involved in waking performance have the potential to modulate cognition through their widespread connections to the cerebral cortex and thus do not act in isolation. For instance, orexin neurons activate regions such as the prefrontal cortex and the basal forebrain cholinergic system, both of which are strongly implicated in normal cognitive function and in age-related cognitive decline.

**Figure 4:**
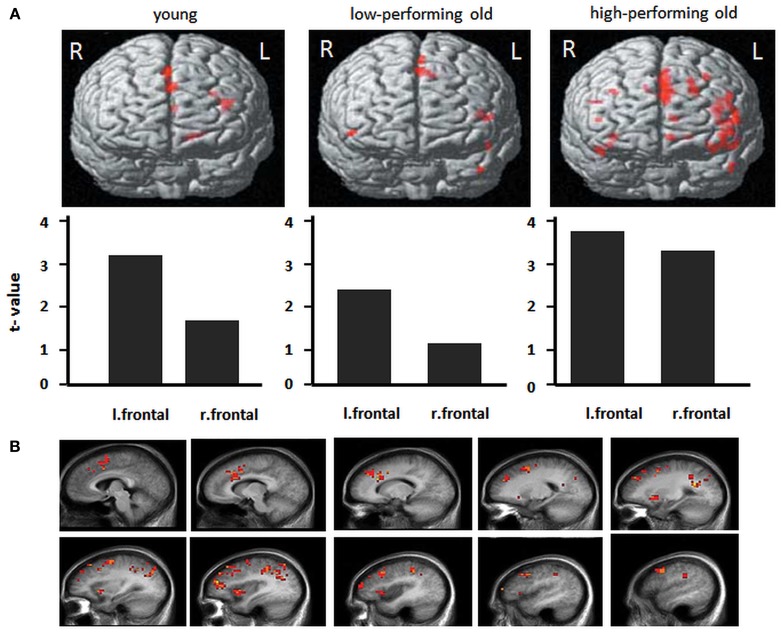
**Frontal activity changes in young and older populations**. **(A)** Activations during a memory-encoding task in young adults, low-performing older adults and high-performing older adults. Low-performing older adults exhibit a similar pattern as do young adults, with lower overall levels of activation. High-performing older adults exhibit greater bilateral activation (with permission from Hedden and Gabrieli, 2004). **(B)** Total sleep deprivation-related patterns of cerebral activation during a verbal learning task. For each of these regions, the memorization of difficult words elicited greater activation after total sleep deprivation than after a night of sleep. Images show left hemisphere slices from 5 to 50 mm [adapted from Drummond et al. (2005b)].

Older age has also been associated with lower blood flow and resting metabolism, particularly in the prefrontal cortex (Meltzer and Francis, 2001) as well as reduced regional brain responses to challenging tasks (see Figure 4; Dennis et al., 2008; see Buckner, 2004; Hedden and Gabrieli, 2004 for a review). Conversely, when performing at optimal levels, older adults present higher functional responses in frontal cortices. Besides quantitative changes, there may be also qualitatively different brain response patterns, such as “over-activation” in an homologous region in the opposite hemisphere from the region typically responsive in young adults, a phenomenon referred to as to “hemispheric asymmetry reduction” in older adults (Eyler et al., 2011). Overall, supplemental recruitment of cerebral structures might reflect compensatory activity or strategic differences with advanced age. A general hypothesis is that increased brain recruitment represents a general response to increasing task difficulty and conveyed global factors that affect aging (Buckner, 2004).

Task-related BOLD activity decreases have been observed in young adults under sleep deprivation, while other studies showed compensatory mechanisms resulting in activity increases (e.g., Drummond et al., 2000; Drummond and Brown, 2001; Chee and Choo, 2004; Habeck et al., 2004; Choo et al., 2005; Mu et al., 2005a,b; Chee et al., 2006; Chuah et al., 2006; Chee and Chuah, 2007; see Chee and Chuah, 2008 for a review). Thus, similarly to what has been hypothesized in the aging literature, it is suggested that increasing task difficulty elicits “compensatory” prefrontal activation in some, but not all studies, as a function of the investigated cognitive domain. Thus sleep deprivation and aging may elicit similar deficits in executive functions due to similar alterations in the prefrontal cortex (e.g., Harrison et al., 2007). Executive functions encompass a series of high-level processes, the main function of which is to facilitate adaptation to new or complex situations when highly practiced cognitive abilities or behaviors no longer suffice (Collette et al., 2006). While the frontal lobes play a major role in executive function (Shallice, 1982), additional posterior cerebral regions, including the parietal lobes (Collette et al., 2006) also play a key role in executive functioning. Horne and colleagues first hypothesized that the waking function of the prefrontal cortex and the frontal predominance of EEG delta activity in sleep may be linked (Horne, 1993; see also Blatter and Cajochen, 2007). Since then, several studies have argued that cognitive functioning related to the prefrontal cortex is particularly vulnerable to sleep loss (Blatter and Cajochen, 2007). However, in a recent report, Tucker et al. (2011) observed heterogeneity in cognitive aspects impaired by sleep deprivation and aging when looking more specifically at the affected cognitive compounds during the performance of executive function tasks, raising questions about the similarity of the involved cognitive processes underlying sleep loss related and age-related modulations in cognitive performance.

## Conclusion and Perspectives

Healthy older individuals may experience twice as much time awake during a night sleep episode than young adults, suggesting that impaired sleep consolidation is associated with aging *per se*, rather than being a by-product of co-ailments linked to aging. Whether age-related sleep disruption derives from either the circadian and/or the homeostatic facet of sleep regulation is still somewhat uncertain. However, we propose that the strength of the circadian/homeostatic interaction on modulating sleep and cognitive processes are weakened in older healthy people. The cortical underpinnings that account for these age-related modulations are virtually unexplored. A combined investigation of these issues at the cerebral level would allow the elaboration of theoretical concepts in order to explain the underlying mechanisms of age-related changes in circadian and homeostatic modulations in cognitive performance. Hence, this leads to the next question: are age-related sleep disruptions a consequence of alterations in the circadian or homeostatic sleep regulation, or is it ultimately caused by an attenuated interaction between the circadian timing system and sleep-wake homeostatic process at the cerebral level? Here we surmise that some of the subcortical structures involved in the generation of circadian wake promotion may at least in part be compromised in healthy aging. Alternatively, upstream from these structures, more integrative cortical areas underlying higher order cognitive behaviors may also be selectively altered with aging. Quantitative evidence for such a hypothesis is building up from the vulnerability of frontal cortical brain regions to both the effects of elevated sleep pressure and aging. Whether there is a direct relationship between age-related decline in cognitive performance and sleep disruption still remains a matter of investigation. Prospective or intervention studies (e.g., sleep extension) would help to elucidate their relationships.

## Conflict of Interest Statement

The authors declare that the research was conducted in the absence of any commercial or financial relationships that could be construed as a potential conflict of interest.
